# Computational Model of Ca^2+^ Wave Propagation in Human Retinal Pigment Epithelial ARPE-19 Cells

**DOI:** 10.1371/journal.pone.0128434

**Published:** 2015-06-12

**Authors:** Iina Vainio, Amna Abu Khamidakh, Michelangelo Paci, Heli Skottman, Kati Juuti-Uusitalo, Jari Hyttinen, Soile Nymark

**Affiliations:** 1 Department of Electronics and Communications Engineering, Tampere University of Technology, Tampere, Finland; 2 Institute of Biosciences and Medical Technology, Tampere University of Technology, Tampere, Finland; 3 Institute of Biosciences and Medical Technology, University of Tampere, Tampere, Finland; Medical University of South Carolina, UNITED STATES

## Abstract

**Objective:**

Computational models of calcium (Ca^2+^) signaling have been constructed for several cell types. There are, however, no such models for retinal pigment epithelium (RPE). Our aim was to construct a Ca^2+^ signaling model for RPE based on our experimental data of mechanically induced Ca^2+^ wave in the *in vitro* model of RPE, the ARPE-19 monolayer.

**Methods:**

We combined six essential Ca^2+^ signaling components into a model: stretch-sensitive Ca^2+^ channels (SSCCs), P_2_Y_2_ receptors, IP_3_ receptors, ryanodine receptors, Ca^2+^ pumps, and gap junctions. The cells in our epithelial model are connected to each other to enable transport of signaling molecules. Parameterization was done by tuning the above model components so that the simulated Ca^2+^ waves reproduced our control experimental data and data where gap junctions were blocked.

**Results:**

Our model was able to explain Ca^2+^ signaling in ARPE-19 cells, and the basic mechanism was found to be as follows: 1) Cells near the stimulus site are likely to conduct Ca^2+^ through plasma membrane SSCCs and gap junctions conduct the Ca^2+^ and IP^3^ between cells further away. 2) Most likely the stimulated cell secretes ligand to the extracellular space where the ligand diffusion mediates the Ca^2+^ signal so that the ligand concentration decreases with distance. 3) The phosphorylation of the IP_3_ receptor defines the cell’s sensitivity to the extracellular ligand attenuating the Ca^2+^ signal in the distance.

**Conclusions:**

The developed model was able to simulate an array of experimental data including drug effects. Furthermore, our simulations predict that suramin may interfere ligand binding on P_2_Y_2_ receptors or accelerate P_2_Y_2_ receptor phosphorylation, which may partially be the reason for Ca^2+^ wave attenuation by suramin. Being the first RPE Ca^2+^ signaling model created based on experimental data on ARPE-19 cell line, the model offers a platform for further modeling of native RPE functions.

## Introduction

Epithelial tissue covers and lines all internal and external body surfaces. These cell layers have multiple functions depending on their location, and many of these functions are controlled by Ca^2+^ activity[1]. Retinal pigment epithelium (RPE), a monolayer of pigmented polarized cells, is crucial for the maintenance of visual functions. Located in the back of the eye between photoreceptors and choriocapillaries, RPE forms a vital part of the blood-retinal barrier (BRB)[2]. The physiology of RPE is tightly coupled with the activity of the various ion channels, such as Ca^2+^ channels that are associated with several important RPE functions including transepithelial transport of ions and water, dark adaption of photoreceptor activity, phagocytosis, secretion, and differentiation[3]. In RPE, as well as in other epithelia, local deformation of the cell membrane initiates a significant Ca^2+^ wave [4–6]. Such deformation of the cell membrane can occur in clinically important pathological conditions such as retinal tear resulting from complications after photodynamic therapy[7], intravitreal bevacizumab injection[8], or intravitreal pegaptanib injection[9]. Intercellular Ca^2+^ signaling is also linked to the initial stages of wound repair: excessive mechanical stimulation causes cell death and thus initiates Ca^2+^ waves that create Ca^2+^ gradients which play an important role in cell migration[1]. In addition, Ca^2+^ waves also regulate the local transepithelial ion transport to maintain the spatial ion gradients across the epithelium[1]. We recently demonstrated in RPE that an easily induced and repeatable Ca^2+^ wave could be produced by mechanical stimulation[5]. This provides an experimental way to study Ca^2+^ activity in the epithelial monolayer.

*In silico* models of various cellular processes are becoming an increasingly important part of biological research, including drug discovery and toxicology studies. The importance of this was recently emphasized in a review of cardiotoxicity testing [10]. Computational models of Ca^2+^ signaling, specifically, have been developed for many cell types including pancreatic and parotid acinar cells[11], astrocytes[12], and hepatocytes[13]. Epithelial Ca^2+^ signaling, however, differs from other cell types because the epithelium forms a highly polarized cell monolayer that comprises organized apical and basal cell membranes. The epithelial cells are tightly connected with tight junctions and gap junctions between the cells[14]. At present, there are only a few epithelial Ca^2+^ signaling models available, for example for the urothelial monolayer[15] and for the airway epithelium[16]. RPE has many unique functions compared to other epithelia as it supports the complex processes of vision. Indeed, in the treatment of many eye diseases, RPE is either the drug target or it hinders drug penetration and provides a barrier between most of the eye and the blood stream. Hence, computational models of the functions of RPE, including Ca^2+^ dynamics, are well warranted.

The aim of this study, therefore, is to provide a deeper understanding of the study of Ca^2+^ activity by introducing a detailed computational model of RPE Ca^2+^ dynamics. The computational model described in this paper is based on our experimental data on a mechanically induced Ca^2+^ wave in ARPE-19 cells, a commercial immortalized human RPE cell line that is widely used to assess RPE cell functions *in vitro* [17–19], regardless of its limitations in cellular morphology, organization and function [20].

The computational model is mostly based on the experimental data of Abu Khamidakh et al. 2013[5]. In addition, the model comprises our new unpublished α-glycyrrhetinic acid (GA)-suramin-treated data. We constructed the model by combining previously published cell Ca^2+^ dynamics model components of P_2_Y_2_ receptors [21], inositol 1,4,5-trisphosphate (IP_3_) receptors [22], ryanodine receptors [23], Ca^2+^ pumps and gap junctions to a new model component of mechanical stretch. Furthermore, we connected the epithelial cells to each other in the model to enable the diffusion of the molecules and propagation of the stretch. We developed the model based on two experimental data sets: the GA-treated data, where gap junctions (GJs) were blocked by α-glycyrrhetinic acid and untreated control data, where GJs define the connections between the cells. The varying conditions the cells are exposed to due to the mechanical stimulation were modeled by defining three location-specific variables: stretch, extracellular ligand concentration, and IP_3_ receptor phosphorylation rate. In addition, we validated the model by simulating the combined blocking effect of GJs and P_2_ receptors by GA and suramin. This way, we obtained the first RPE Ca^2+^ signaling model, and we could reveal a deeper understanding of Ca^2+^ activity.

## Materials and Methods

### Experimental data

**Fig 1:**
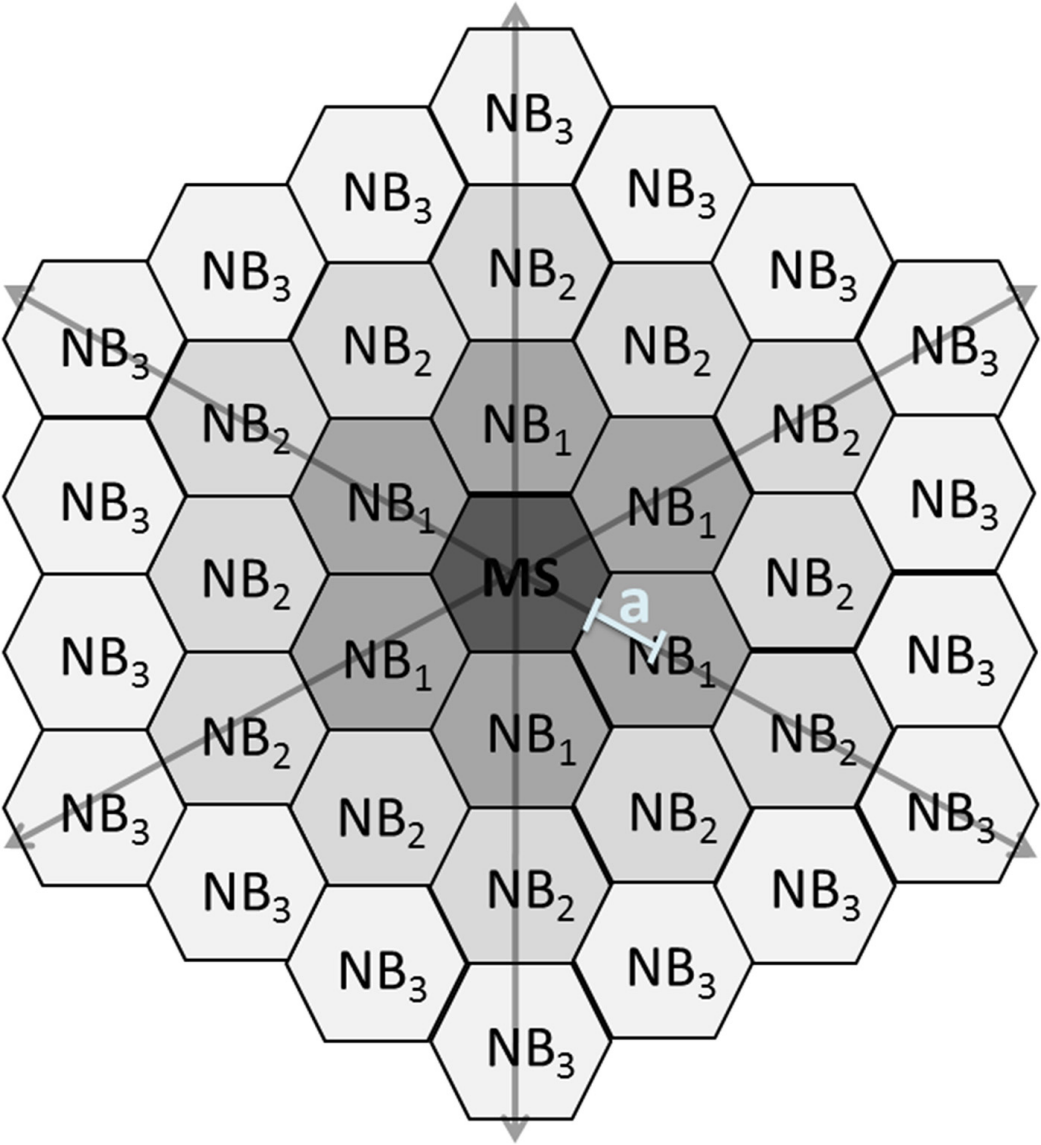
Numbering of the cell layers. Schematic representation of the location of the mechanically stimulated (MS) cell with respect to the neighboring (NB) cell layers: NB_1_ is the first layer which is in direct contact with the MS cell; NB_2_ is the second layer which is in direct contact with NB_1_, and so forth. White line segment marks an apothem (a) of a hexagon.

In this study, the experimental data of Abu Khamidakh et al. 2013[5] was complemented with new experimental data. Passage numbers for confluent cultures of human RPE immortalized cells (ARPE-19 cell line [ATCC Manassas, VA, U.S.A.]) were p. 23, 24, 30 for GA-treated dataset, p. 23, 24, 28, 30 for control data set and p. 29, 30, 31 for GA-suramin-treated data set. These ARPE-19 cultures were used for Ca^2+^ imaging, by loading them with the Ca^2+^-sensitive dye fura-2-acetoxymethyl ester. Single cell mechanical stimulation, membrane perforation of one cell, was induced with a glass micropipette. The intracellular Ca^2+^ concentration transient travelled over the ARPE-19 monolayer starting from the mechanically stimulated (MS) cell, and spreading to the neighboring (NB) cells (Fig 1). The NB cells immediately surrounding the MS cell were defined as the first NB cell layer (NB layer 1 = NB_1_); cells immediately surrounding the first layer were defined as the second NB layer (NB layer 2 = NB_2_) and so on. The ratio of the emitted fluorescence intensities resulting from excitation at 340 and 380 nm (F_340_/F_380_) was determined for each cell after background correction. Normalized fluorescence (NF), which reflects the changes in intracellular Ca^2+^ concentration, was then obtained by dividing the fluorescence value by the mean fluorescence value before the mechanical stimulation.[5] The experimental work produced data in NF units. The computational model, however, is presented in absolute calcium concentrations. Due to the lack of absolute reference we consider the model predictions only relative.

Three data sets were simulated with the model. Firstly, in the GA-treated data set the gap junctions (GJs) were blocked by α-glycyrrhetinic acid (GA) (Sigma-Aldrich, St. Louis, MO, USA). Secondly, the model was verified with an untreated control data set that was based on the previous model—only the GJ model component was added. Thirdly, the model was applied to predict a combined blocking effect of GA and P_2_ receptor blocker suramin (Sigma-Aldrich) with GA-suramin-treated data set. Each data set was averaged from at least three separate experiments.

The experiments with GA-suramin-treated ARPE-19 cells were not included in the original paper of Abu Khamidakh et al. 2013[5]. The experimental details concerning the ARPE-19 cells as well as the experimental solutions, infrastructure, and protocols are presented in[5] with the following exception: the cells were incubated in a solution containing 30μM GA (incubation time 30 min) and 50μM suramin (incubation time 25 min) prior to mechanical stimulation. To receive representative data for each NB layer, the raw data was averaged so that the NF graphs were aligned by the starting time of mechanical stimulation, and the mean values were calculated for each NB with a one-second sampling rate. This was previously done for the control data set[5], but the averaging was performed also here for the GA-treated and GA-suramin-treated data sets.

#### Indirect immunofluorescence staining

ARPE-19 cells (p. 24, 27, 44, three cover slips from each passage) were cultured on glass coverslips for two days. For immunofluorescence staining, the samples were washed three times with PBS and fixed for 15 min with 4% paraformaldehyde (pH 7.4; Sigma-Aldrich) at room temperature (RT). After three subsequent washes with PBS, the samples were permeabilizing by a 15 min incubation in 0.1% Triton X-100 in PBS (Sigma-Aldrich) at RT. This was followed again by three PBS washes, after which the samples were incubated with 3% bovine serum albumin (BSA; Sigma-Aldrich) at RT for 1 h. Primary antibody Zonula Occludens (ZO-1) 1:100 (33–9100, Life Technologies) was diluted in 3% BSA PBS and incubated for 1 h at RT. Samples were then washed four times with PBS, and followed by 1h incubation at RT with secondary antibody donkey anti-mouse Alexa Fluor 568 (A10037, Life Technologies) diluted 1:400 in 3% BSA in PBS. The washes with PBS were repeated again and nuclei were stained with 4′, 6′ diamidino-2-phenylidole (DAPI) included in the mounting medium (P36935, Life Technologies).

#### Confocal microscopy and image processing

Zeiss LSM780 LSCM on inverted Zeiss Cell Observer microscope (Zeiss, Jena, Germany) with Plan-Apochromat 63x/1.4 oil immersion objective was used for confocal microscopy. Voxel size was set to x = y = 66nm and z = 200nm, pixel stacks were set to 1024x1024, and approximately 50–80 slices were acquired with line average of 2. DAPI and Alexa-568 were excited with 405nm and 561nm lasers and detected with emission windows of 410–495nm and 570–642nm, respectively. The images saved in czi format were processed with ImageJ (Rasband, W.S., ImageJ, U. S. National Institutes of Health, Bethesda, Maryland, USA, http://imagej.nih.gov/ij/, 1997–2014.) and assembled using Adobe Photoshop CS6 (Adobe Systems, San Jose, USA).

### Construction of the model

**Fig 2:**
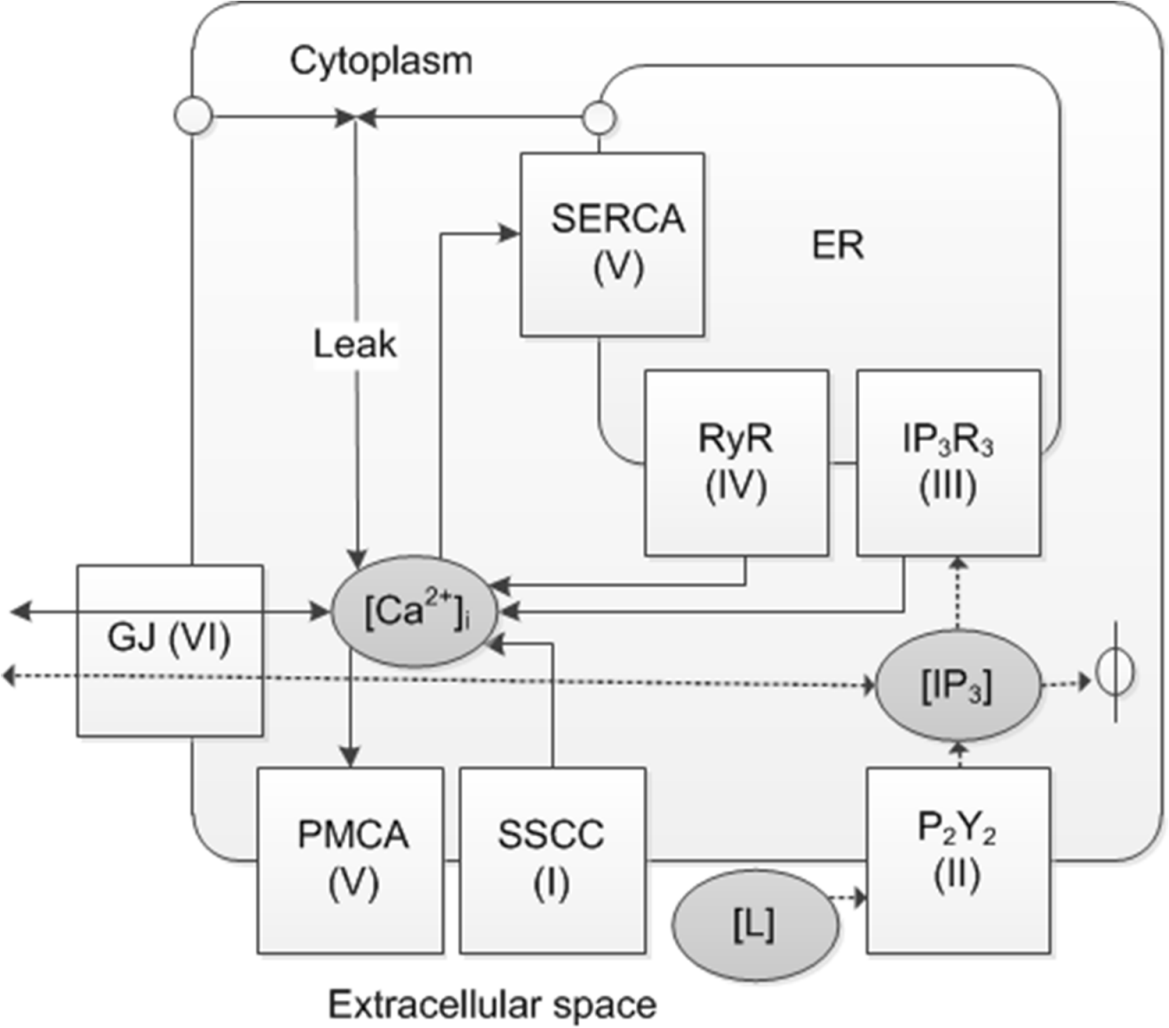
Schematic diagram of the Ca^2+^ signaling model. Solid arrows represent Ca^**2+**^ fluxes and dashed arrows IP_3_ dynamics. Roman numerals denote the model components I-VI. Abbreviations: [Ca^**2+**^]_i_ = cytoplasmic Ca^**2+**^ concentration, [L] = extracellular ligand concentration, [IP_3_] = cytoplasmic IP_3_ concentration, SSCC = stretch-sensitive Ca^**2+**^ channel, P_2_Y_2_ = purinergic receptor type P_2_Y_2_, IP_3_R_3_ = IP_3_ receptor type 3, RyR = ryanodine receptor, SERCA = sarco/endoplasmic reticulum Ca^**2+**^ ATPase, PMCA = plasma membrane Ca^**2+**^ ATPase, Leak = combinatory Ca^**2+**^ leak from the extracellular space and the endoplasmic reticulum (ER), GJ = gap junction, ϕ = degradation.

The Ca^2+^ model was constructed by combining six subcellular model components that included the stretch component designed in this study and the P_2_Y_2_ receptor models of Lemon et al. 2003[21], the IP_3_ receptor type 3 (IP_3_R_3_) of LeBeau et al. 1999[22], and the ryanodine receptor (RyR) of Keizer & Levine 1996[23]. The GJ model component connected the neighboring cells. These model components with corresponding numbering and their rationale, hypothesized Ca^2+^ wave propagation mechanisms as well as model equations (see chapter Detailed model equations) that were used for the NB layers and data sets are summarized in Table 1. The basis for the mathematical implementation is presented in Fig 2 as a schematic model.

**Table 1:** Model design.

Mechanism	Number	Component	Rationale	Equations	NB layer	Data set
Mechanical stimulus applied to MS cell may stretch ARPE-19 cells near the site of stimulation [5] resulting in the opening of SSCCs that conduct Ca^2+^ from the extracellular space to the cytoplasm. It is shown that ARPE-19 cells can secrete ligand to the extracellular space as a response to stimuli [38].	I	Stretch-sensitive Ca^2+^ channel (SSCC)	Cultured rat RPE expresses SSCCs on plasma membrane [4,53]. In ARPE-19 [Ca^2+^]_i_ wave was seen in NB_1_-NB_4_ layers even when the ER was depleted[5], indicating a Ca^2+^ influx mechanism independent of the ER, possibly the SSCCs.	7–9^a^	1–4	GA-treated, Control, GA-suramin-treated
The ligand, likely ATP or UTP [3,30,38,54], interacts with G-protein coupled purinergic receptor type P_2_Y_2_ on the cell membrane leading to the production of inositol 1,4,5-trisphosphate (IP_3_) to the cytoplasm in a ligand concentration dependent manner.	II	Purinergic P_2_Y_2_ receptor (P_2_Y_2_)	The presence of P_2_Y_2_ receptors has been shown in cultured human RPE[30], bovine and human fetal RPE as well as in Long-Evans rats[55]	10–16^b^	1–10	GA-treated, Control, GA-suramin-treated^e^
IP_3_ diffuses across the cytoplasm to the endoplasmic reticulum (ER), where it interacts with IP_3_R_3_ resulting in a release of Ca^2+^ from the ER[22].	III	IP_3_ receptor type 3 (IP_3_R_3_)	Currently there is no direct evidence about the subtype of IP_3_R expressed in ARPE-19. Hence, the data from other epithelia [56], [57], and[58] and an epithelial model [16] was utilized to choose the subtype 3 (IP_3_R_3_).	17–21^c^	1–10	GA-treated, Control, GA-suramin-treated
As the cytoplasmic Ca^2+^ concentration increases, RyRs become activated releasing more Ca^2+^ to the cytoplasm from the ER[23].	IV	Ryanodine receptor (RyR)	RyRs, locating on the membrane of ER participate in Ca^2+^ signaling in rat RPE[4], and ARPE-19[51].	22–24^d^	1–10	GA-treated, Control, GA-suramin-treated
The cytoplasmic Ca^2+^ concentration is decreased by the pumping activities of SERCA and PMCA. IP_3_ is degraded in the cytoplasm. Ca^2+^ leak currents maintain the cytoplasmic Ca^2+^ baseline level.	V	Sarco/endoplasmic reticulum ATPase (SERCA), plasma membrane Ca^2+^ ATPase (PMCA), Leak	The presence of SERCA has been shown by blocking it to deplete the ER from Ca^2+^ in ARPE-19 cells[5] and rat RPE[53]. PMCA has been identified on the plasma membrane of cultured human RPE[59].	25	1–10	GA-treated, Control, GA-suramin-treated
GJs form intercellular connections between neighboring cells allowing diffusion of Ca^2+^ and IP_3_ between the NB layers.	VI	Gap junction (GJ)	GJs form intercellular connections in ARPE-19[5], and rat RPE[4,60] enabling Ca^2+^ wave to spread over the monolayer.	26–28	1–10	Control

^a^Designed in this study

^b^Lemon et al. 2003[21]

^c^LeBeau et al. 1999[22]

^d^Keizer & Levine 1996[23]

^e^Parameter values of k_p_ modified

Hypothesized mechanisms and model components for Ca^2+^ wave propagation after mechanical stimulation with corresponding equations, NB layers, and data sets.

### Parameters and parameterization

The model parameters are represented in Table 2 and the parameters specific for each NB layer in Table 3. Most of the parameters were adopted from the models of Lemon et al. 2003[21], LeBeau et al. 1999[22], and Keizer & Levine 1996[23]. Typically, the volumes of ARPE-19 cells[5,24,25] and RPE cells[26,27] are variable. The cell width was approximated to be 14μm from the corner-to-corner of a hexagon and the height was 12μm[5].The cytoplasmic volume was approximated to be about 70% of the total cell volume[28]. Thus, a cytoplasmic volume (v) of 1.07 10^–15^ m^3^ was used in the simulations. The initial values, the values at time of mechanical stimulation, were taken mostly from the model of Lemon et al. 2003[21]. The initial value 0.12μM for intracellular Ca^2+^ concentration ([Ca^2+^]_i_) is an arbitrary value approximating the baseline Ca^2+^ concentration determined from GA-treated data set for NB_5_-NB_10_ layers using Matlab SimBiology Toolbox.

**Table 2:** Constant parameters and initial conditions.

Parameter	Description	Value	Reference
**I Stretch-sensitive Ca** ^**2+**^ **channels (SSCCs)**			
k_SSCC_	Maximal SSCC flux rate	1.025 μM s^-1^	fitted
k_f_	SSCC forward rate constant	0.1382 s^-1^	fitted
k_b_	SSCC backward rate constant	0.04027 s^-1^	fitted
k_θ_	Stretch-relaxation parameter	0.08105 s^-1^	fitted
**II Metabotropic receptor P**_**2**_ **Y**_**2**_			
L_0_	Bolus extracellular ligand concentration at x = 0μm	1310 μM	fitted
D_ATP_	Diffusion coefficient of A	236 μm^2^ s^-1^	[61]
[R_T_]	Total number of P_2_Y_2_ receptors	2∙10^4^	[62]
K_1_	Unphosphorylated receptor dissociation constant	5 μM	[21]
K_2_	Phosphorylated receptor dissociation constant	100 μM	[21]
k_r_	Receptor recycling rate	1.75∙10^–4^ s^-1^	[21]
k_p_	Receptor phosphorylation rate	0.03 s^-1^	[21]
k_e_	Receptor endocytosis rate	6∙10^–3^ s^-1^	[21]
ξ	Fraction of mobile receptors	0.85	[21]
[G_T_]	Total number of G-protein molecules	1∙10^5^	[63]
k_deg_	IP_3_ degradation rate	1.25 s^-1^	[64]
k_a_	G-protein activation rate	0.017 s^-1^	[21]
k_d_	G-protein deactivation rate	0.15 s^-1^	[21]
[(PIP_2_)_T_]	Total number of PIP_2_ molecules	5.0∙10^4^	[21]
r_r_	PIP_2_ replenishment rate	0.015 s^-1^	[21]
δ	G-protein intrinsic activity parameter	1.238∙10^–3^	[21]
K_3_	Dissociation constant for Ca^2+^ binding to PLC	0.4 μM	[21]
α	Effective signal gain parameter	2.781∙10^–5^ s^-1^	[21]
N_a_	Avogadro's constant	6.02252∙10^23^	
v	Volume of the cytoplasmic space	1.07∙10^–15^ m^3^	see text
**III IP**_**3**_ **receptor type 3 (IP**_**3**_ **R**_**3**_ **)**			
α_1_	Maximum rate of k_1_	40 μM s^-1^	[22]
β_1_	[Ca^2+^]_i_ for half-maximal k_1_	0.8 μM	[22]
k_-1_	Rate of O to S transition	0.88 s^-1^	[22]
k_2_	Rate of O to I_1_ transition	0.5 s^-1^	[22]
k_3_	Rate of I_1_ to S transition	0.5 s^-1^	[22]
β_4_	[IP_3_] for half-maximal k_4_	0.01 μM	[22]
k_5_	Rate of I_2_ to S transition	0.02 s^-1^	[22]
$k_{IP_{3}R_{3}}$	Maximum IP_3_R_3_ flux rate	155.8 μM s^-1^	fitted
**IV Ryanodine receptor (RyR)**			
K_a_	Keizer & Levine dissociation constant	0.37224 μM	[23]
K_b_	Keizer & Levine dissociation constant	0.63601 μM	[23]
K_c_	Keizer & Levine dissociation constant	0.05714 μM	[23]
k_RyR_	Maximum RyR flux rate	16.04 μM s^-1^	fitted
**V Ca** ^**2+**^ **pumps and leak current**			
V_Pump_	Maximal pump rate	5.341 μM s^-1^	fitted
K_Pump_	[Ca^2+^]_i_ for half-maximal V_Pump_	0.5030 μM	fitted
J_Leak_	Ca^2+^ leak current	0.1450 μM s^-1^	fitted
**VI Gap junctions (GJ)**			
$D_{Ca^{2+}}$	Diffusion coefficient of Ca^2+^ through GJs	512.7 μm^2^ s^-1^	fitted
$D_{IP_{3}}$	Diffusion coefficient of IP_3_ through GJs	913.9 μm^2^ s^-1^	fitted
$In_{Ca^{2+}}$	Ca^2+^ input to NB1	-0.003320 μM s^-1^	fitted
$In_{IP_{3}}$	IP_3_ input to NB1	0.5771 μM s^-1^	fitted
$Out_{Ca^{2+}}$	Ca^2+^ output from NB10	0 μM s^-1^	see text
$Out_{IP_{3}}$	IP_3_ output from NB10	0 μM s^-1^	see text
**Initial conditions (time 0s)**			
[R^S^]	Total number of unphosphorylated surface receptors	17000	[21]
[R^S^_p_]	Total number of phosphorylated surface receptors	0	[21]
[G]	Basal number of G-protein molecules	14	[21]
[IP_3_]	Basal IP_3_ concentration	0.01 μM	[21]
[PIP_2_]	Basal number of PIP_2_ molecules	49997	[21]
[Ca^2+^]_i_	Basal cytoplasmic Ca^2+^ concentration	0.12 μM	see text

Most of the parameters were taken from the models of Lemon et al. 2003[21] for P_2_Y_2_ receptor, LeBeau et al. 1999[22] for IP_3_R_3_, and Keizer & Levine 1996[23] for RyR. Reference ‘fitted’ means that the parameter was optimized in this study.

**Table 3:** Location-dependent parameters with respect to the MS cell.

Parameter	Description	Equation	Range
x	Distance from the MS cell centre	1	From 12.12 μm (NB1) to 121.24 μm (NB10)
θ	Stretch	2 (exponential decay)	From 0.096 (NB1) to 1.014 10^–6^ (NB10)
L	Extracellular ligand concentration	3 (exponential decay)	From 26.14 μM (NB1) to 2.61 μM (NB10)
α_4_	IP_3_R_3_ phosphorylation rate	4 (exponential rise)	From 0.0413 s^-1^ (NB1) to 0.1548 s^-1^ (NB10)
		5 (exponential rise)	From 0.0333 (NB1) to 0.1503 (NB10)
_$J_{GJ,Ca^{2+}}$_	Ca^2+^ flux through GJs	26	From 0.049 μM s^-1^ (NB1→NB2) to 1.8 10^–6^ μM s^-1^ (NB9→NB10)
$J_{GJ,IP_{3}}$	IP_3_ flux through GJs	27	From 0.407 μM s^-1^ (NB1→NB2) to 0.022 μM s^-1^ (NB9→NB10)
A	Area of the cell membranes connecting NB layers	28	From 1512 μm^2^ (NB1) to 10584 μm^2^ (NB10)

The rest of the parameters were fitted with Matlab SimBiology Parameter Fit Task: First, the parameter values, excluding SSCC and GJ model components, were fitted with GA-treated data set in NB5 layer. This layer has in general the largest Ca^2+^ response from those NB layers that do not experience any stretch due to mechanical stimulation, according to our assumption. Secondly, the SSCC model component parameters, excluding the location-specific stretch (θ) parameter (see below), were fitted with the same GA-treated data set in NB1 layer that is assumed to have the largest stretch. These values were then used in all simulations for all data sets and NB layers. For the control data set with gap junctions, all other parameters were kept unchanged but the GJ related diffusion parameters, $D_{Ca^{2+}}$, $D_{IP_{3}}$, $In_{Ca^{2+}}$ and $In_{IP_{3}}$, were fitted using NB1 layer. As a boundary condition we assumed that there is no outflow of IP_3_ and Ca^2+^ outside the epithelium, thus $Out_{IP_{3}}$ and $Out_{Ca^{2+}}$were assigned to be zero.

### Location-dependent parameters

Three parameters were assumed to vary according to the location of the cell with respect to the MS cell: stretch (θ) activating the stretch-sensitive Ca^2+^ channels (SSCCs), the extracellular ligand concentration ([L])[6,16,29,30], and the phosphorylation rate of IP_3_R_3_ (α_4_)[22]. Ca^2+^ concentration was modeled separately in each NB layer. The distance (x) defines the distance of the NB layer from the MS cell centre that was calculated using the idealized hexacon RPE cell architecture (Fig 1) as

$$x=a2n=\frac{s}{2\tan\left(\frac{180}{6}\right)}2n,$$ (1)

where a is an apothem of the hexagon, 6 is the number of corners in the hexagon, s = 7μm is the length of the hexagon side and n = 1, 2, 3…10 according to the NB layer numbering.

The stretch component was present in cell layers NB1-NB4. Stretch (θ) was parameterized in the GA-treated data set separately for each NB layer. The obtained parameters resulted in an exponentially decaying function corresponding to the decay of an amplitude envelope of a damped wave in a membrane [31]. This function was then used for modeling the stretch

$$\theta =0.3426e^{-0.105x},$$ (2)

where x is the distance from the MS cell (R^2^ = 0.9878).

Ligand diffusion in the extracellular space is modelled according to thin film solution to Fick’s diffusion law [32] as follows describing the ligand concentration (L) as a function of time (t)

$$L(x,t)=\frac{L_{0}}{\sqrt{4\pi D_{ATP}t}}e^{-x^{2}/\left(4Dt\right)},$$ (3)

where L_0_ is the initial bolus ligand concentration above the MS cell (at x = 0), D_ATP_ is the diffusion coefficient for ATP, and x describes the NB layer distance from the central MS cell.

IP_3_R_3_ phosphorylation rate (α_4_) used in Eq 21 was fitted separately for each NB layer in GA-treated and control data sets, which resulted in shallowly rising exponential functions with respect to the distance of the cell from the MS cell (x). The equation for GA-treated data set (R^2^ = 0.9740) is

$$\alpha_{4}=0.0357e^{0.0121x}$$ (4)

and for control data set (R^2^ = 0.9798)

$$\alpha_{4}=0.0282e^{0.0138x}.$$ (5)

Similarly to θ and L, these functions were then used in simulations instead of values from separate fits.

### Model simulations

The parameters were fitted with Matlab SimBiology (R2012a, The MathWorks, Natick, MA) to the experimental data using Parameter Fit task, where the maximum iterations was 100. The solver type was ode45 (Dormand-Prince) and the error model was constant error model. The time step in the simulations was set to ∆t = 0.1 seconds.

### Sensitivity analysis

Sensitivity analysis was performed to evaluate the uncertainty of selected parameters that were fitted in this study (parameters $k_{IP_{3}R_{3}}$, k_RyR_, V_Pump_, K_Pump_, J_Leak_, $In_{IP_{3}}$, $In_{Ca^{2+}}$, $D_{IP_{3}}$ and $D_{IP_{3}}$from Table 2) or behaved as location-specific parameters (parameters θ, L and α_4_ from Table 3). Values of these parameters were changed -25%, -10%, 0%, +10% and +25% in the model including all the model components I-VI for the control data set. The influence of these parameter were studied for NB layers NB1, NB5 and NB10 concentrating on the following features of the Ca^2+^ wave: peak amplitude, time to peak, Ca^2+^ wave width at half maximum, and Ca^2+^ concentration at the end of the Ca^2+^ wave (at 90 seconds’ time point).

### Model prediction of drug effect: suramin

With the model, we investigated the mechanism by which suramin influences the Ca^2+^ waves in ARPE-19 cells. First, we compared the peak amplitude, time to peak, Ca^2+^ wave width at half maximum, and Ca^2+^ concentration in the end of the Ca^2+^ wave at 90 seconds’ time point between two experimental data sets: GA-treated and GA-suramin-treated data sets. Second, we made sensitivity analysis about the behaviour of P_2_Y_2_ receptor regulation parameters (K_1_, K_2_, k_r_, k_p_, k_e_, ξ), since suramin is a known unspecific antagonist of P_2_ receptors. Suramin has also been suggested to disrupt the coupling between the receptor in the cell membrane and the G-protein by blocking the association of the G-protein α and βγ subunits[33]. Hence, the G-protein cascade parameters k_a_, k_d_ and δ were also evaluated. The sensitivity analysis was done in the model for GA-treated data set (including model components I-V) in NB1, NB5 and NB10 layers. The parameter values were changed in the model by -25% and +25% in order to compare the effects of parameter modifications to the observed differences in the experimental data between GA-treated and GA-suramin-treated data sets. All other parameters were kept unchanged. Third, based on the results of this approach, the model was fitted to the GA-suramin-treated data set by refitting those P_2_Y_2_ receptor and G-protein cascade parameters that were observed to change the Ca^2+^ curve similarly to the differences seen in the experimental data between GA-treated and GA-suramin-treated data sets. This was done with Matlab SimBiology Parameter Fit task for each NB layer.

### Detailed model equations

Time dependent changes in intracellular Ca^2+^ concentration [Ca^2+^]_i_ are presented in the model as a combination of Ca^2+^ fluxes

$$\frac{d{\left[Ca^{2+}\right]}_{i}}{dt}=J_{SSCC}+J_{IP_{3}R_{3}}+J_{RyR}-J_{Pump}+J_{Leak}+J_{GJ,Ca^{2+}},$$ (6)

where the subscripts indicate the source of the flux: stretch-sensitive Ca^2+^ channels (J_SSCC_), inositol 1,4,5-trisphosphate (IP_3_) receptor type 3 ($J_{IP_{3}R_{3}}$), and ryanodine receptor (J_RyR_). J_Pump_ combines the Ca^2+^ pumping functions of sarco/endoplasmic reticulum ATPase (SERCA) and the plasma membrane Ca^2+^ ATPase (PMCA). Leak Ca^2+^ current (J_Leak_) describes the total leakage from the extracellular space and the endoplasmic reticulum (ER) to the cytoplasm. $J_{GJ,Ca^{2+}}$is the Ca^2+^ flux through gap junctions.

#### I Stretch-sensitive Ca^2+^ channels (SSCCs)

**Fig 3:**
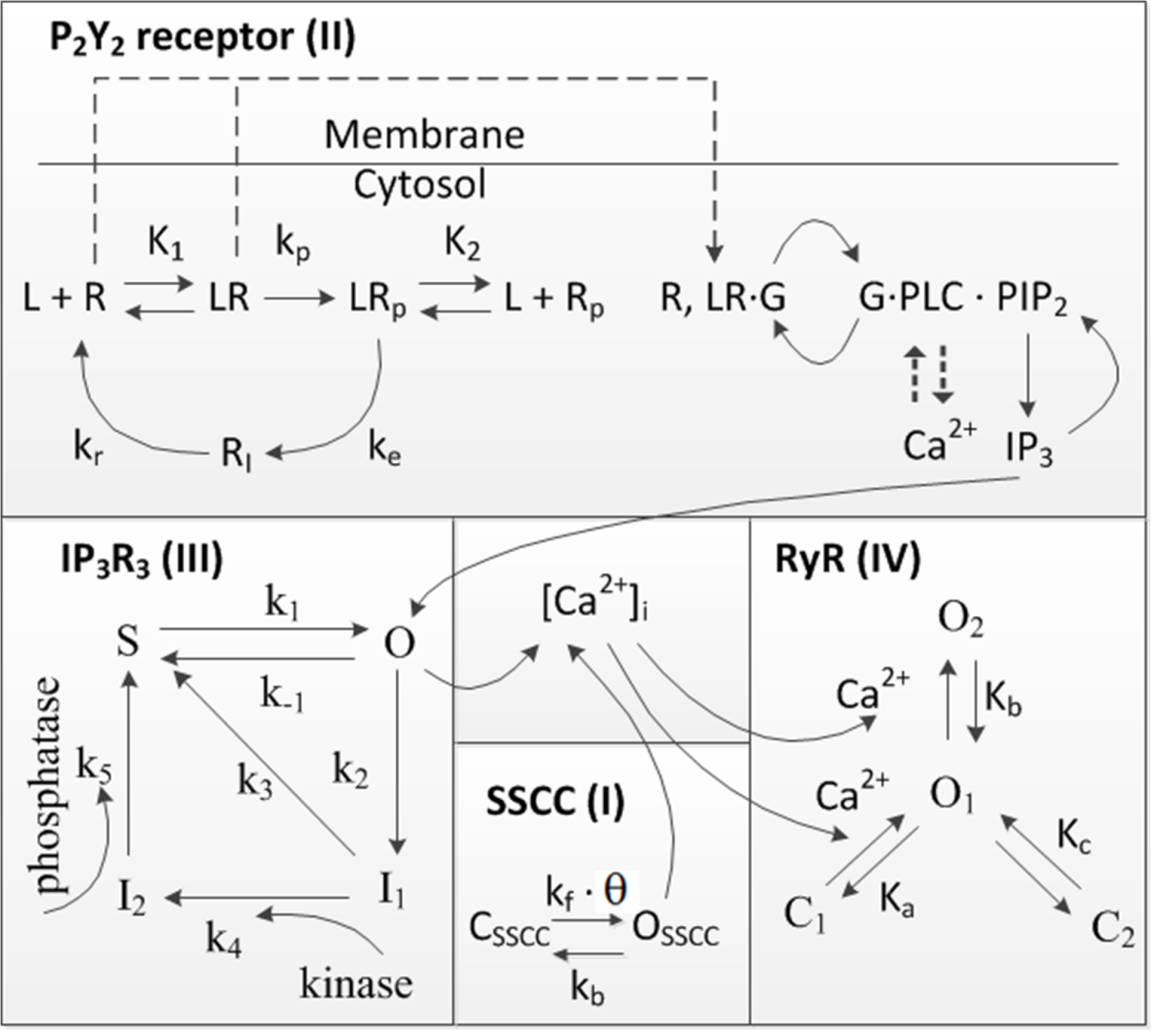
Kinetic diagram. Kinetics of the model component I (SSCC) were combined with the kinetics of model components II-IV from the original models of the P_2_Y_2_ receptor[21], IP_3_R_3_[22], and RyR[23].

Stretch-sensitive Ca^2+^ channels (SSCCs) on the cell membrane are activated, when exposed to mechanical stimulation. Their closure is caused either by relaxation in the mechanical force or by their adaption to that mechanical force[34]. The SSCC model is described with Eqs 7–9. In this study, a model for SSCCs was developed according to the kinetic diagram shown in Fig 3, where C_SSCC_ describes the proportion of the channels in the closed state. O_SSCC_ is the proportion of SSCCs in the open state defined as

$$\frac{dO_{SSCC}}{dt}=\theta k_{f}-\left(\theta k_{f}+k_{b}\right)O_{SSCC},$$ (7)

where k_f_ is the forward rate constant and k_b_ is the backward rate constant. Ca^2+^ flux via SSCCs (J_SSCC_) is expressed as

$$J_{SSCC}=k_{SSCC}O_{SSCC},$$ (8)

where k_SSCC_ is the maximum Ca^2+^ flux rate via SSCCs. Parameter θ is dimensionless, and describes the quantity of stretch induced at the time of mechanical stimulation, which then decreases with time

$$\frac{d\theta }{dt}=-k_{{}_{\theta }}\theta ,$$ (9)

according to a stretch-relaxation parameter k_θ_.

#### II Purinergic receptor P_2_Y_2_

The agonist-induced activation of the second messenger system, here P_2_Y_2_, is represented by Eqs 10–16[21].The kinetic diagram for the P_2_Y_2_ receptor is presented in Fig 3. Some of the ligand-bound P_2_Y_2_ receptors on the cell surface are phosphorylated irreversibly at rate k_p_, which causes desensitization of the receptors. Phosphorylated receptors are internalized at a rate k_e_, and these internalized receptors are then dephosphorylated and recycled back to the surface at rate k_r_. G-proteins can only be activated by the unphosphorylated P_2_Y_2_ receptors [R^S^] defined by

$$\frac{d\left[R^{S}\right]}{dt}=k_{r}\left[R_{T}\right]-\left(k_{r}+\frac{k_{p}\left[L\right]}{K_{1}+\left[L\right]}\right)\left[R^{S}\right]-k_{r}\left[R_{p}^{S}\right],$$ (10)

where [R_T_] denotes the total number of surface receptors, K_1_ is the dissociation constant for unphosphorylated receptors, and [L] is the extracellular ligand concentration. The total number of phosphorylated surface receptors $\left[R_{p}^{S}\right]$ is

$$\frac{d\left[R_{p}^{S}\right]}{dt}=\left[L\right]\left(\frac{k_{p}\left[R^{S}\right]}{K_{1}+\left[L\right]}-\frac{k_{e}\left[R_{p}^{S}\right]}{K_{2}+\left[L\right]}\right),$$ (11)

where K_2_ is the dissociation constant for phosphorylated receptors. The binding of the ligand to the G-protein coupled receptor P_2_Y_2_ results in a cascade of events leading to the activation of enzyme phospholipase C (PLC). This enzyme then hydrolyses the phosphatidylinositol 4,5-bisphosphate (PIP_2_) to IP_3_. The activation rate (k_a_) of the G-protein is proportional to two ratios: the ratio of the activities of the ligand unbound and bound receptor species (δ), and the ratio of the number of ligand bound receptors and the total number of receptors (p_r_). Denoting the deactivation of G-protein to occur at a deactivation rate of k_d_, the equations for the amount of Gα∙GTP labeled as [G] as well as for the ratio p_r_ can be expressed as

$$\frac{d\left[G\right]}{dt}=k_{a}\left(\delta +p_{r}\right)\left(\left[G_{T}\right]-\left[G\right]\right)-k_{d}\left[G\right],$$ (12)

and

$$p_{r}=\frac{\left[L\right]\left[R^{S}\right]}{\xi \left[R_{T}\right]\left(K_{1}+\left[L\right]\right)}.$$ (13)

Equation for the concentration of IP_3_ is

$$\frac{d\left[IP_{3}\right]}{dt}=r_{h}N_{a}{}^{-1}v^{-1}\left[PIP_{2}\right]-k_{\deg}\left[IP_{3}\right]+J_{GJ,IP_{3}},$$ (14)

where k_deg_ is the degradation rate of IP_3_ and $J_{GJ,IP_{3}}$ is the IP_3_ flux through gap junctions. The rate coefficient for PIP_2_ hydrolysis (r_h_) includes the effective signal gain parameter (α) and the dissociation constant for Ca^2+^ binding to PLC (K_3_) that can be expressed as

$$r_{h}=\alpha \left(\frac{{\left[Ca^{2+}\right]}_{i}}{K_{3}+{\left[Ca^{2+}\right]}_{i}}\right)\left[G\right]$$ (15)

Replenishment of PIP_2_ is required for IP_3_ production to be maintained over sustained periods of agonist stimulation. The equation for the number of PIP_2_ molecules [PIP_2_] is

$$\frac{d\left[PIP_{2}\right]}{dt}=-\left(r_{h}+r_{r}\right)\left[PIP_{2}\right]-r_{r}N_{a}v\left[IP_{3}\right]+r_{r}\left[{\left(PIP_{2}\right)}_{T}\right],$$ (16)

where r_r_ represents the PIP_2_ replenishment rate and [(PIP_2_)_T_] the total number of PIP_2_ molecules.[21]

#### III IP_3_ receptor type 3 (IP_3_R_3_)

The IP_3_ receptor type 3 (IP_3_R_3_) function is represented by the Eqs 17–21[22]. The kinetic diagram for IP_3_R_3_ is shown in Fig 3. The IP_3_-induced release of Ca^2+^ from the ER through IP_3_R_3_ ($J_{IP_{3}R_{3}}$) is

$$J_{IP_{3}R_{3}}=k_{IP_{3}R_{3}}O^{4},$$ (17)

where $k_{IP_{3}R_{3}}$ is the maximum rate of Ca^2+^ release, and IP_3_R_3_ comprises four subunits that all must be in the open state (O) for the receptor to conduct. The steady-state proportion of open receptors (O) is

$$O=\frac{\phi \left[IP_{3}\right]}{\frac{k_{-1}+k_{2}}{k_{1}}\phi +\left[IP_{3}\right]},$$ (18)

Where *ϕ* function controls the sensitivity of IP_3_R_3_ to [IP_3_], and it can be expressed as

$$\phi =\frac{1}{1+\frac{k_{{}_{2}}}{k_{3}+k_{4}}\left(1+\frac{k_{4}}{k_{5}}\right)},$$ (19)

with rate coefficients k_-1_, k_2_, k_3_, and k_5_ being constants. Coefficient k_1_ describes a rate for IP_3_R_3_ transition from shut state (S) to open state (O)

$$k_{1}=\frac{\alpha_{1}{\left[Ca^{2+}\right]}_{i}{}^{3}}{\beta_{1}{}^{3}+{\left[Ca^{2+}\right]}_{i}{}^{3}},$$ (20)

where constant α_1_ is the maximum rate of S to O transition, and β_1_ is the [Ca^2+^]_i_ at which the rate is half of its maximum. Coefficient k_4_ expresses the rate for IP_3_R_3_ from the first inactivated state (I_1_) to the second inactivated state (I_2_). It can be expressed as

$$k_{4}=\frac{\alpha_{4}\left[IP_{3}\right]}{\beta_{4}+\left[IP_{3}\right]},$$ (21)

where the I_1_ to I_2_ transition is agonist specific and involves a phosphorylation of IP_3_R_3_ by kinase activity. This is defined by parameter α_4_ that denotes the maximum rate of I_1_ to I_2_ transition, while β_4_ denotes the value of [IP_3_] at which the rate is half maximal.[22]

#### IV Ryanodine receptor (RyR)

The ryanodine receptor (RyR) dynamics were modeled by Keizer & Levine 1996[23] with Eqs 22–24. In Fig 3 the kinetic diagram for RyR is illustrated. The Ca^2+^ release from the ER through RyR (J_RyR_) is defined by the maximum RyR flux rate (k_RyR_) multiplied by the open probability (P_RyR_) as

$$J_{RyR}=k_{RyR}P_{RyR}$$ (22)

where

$${}_{P_{RyR}=\left(\frac{w^{\infty }\left(1+{\left(\frac{{\left[Ca^{2+}\right]}_{i}}{K_{b}}\right)}^{3}\right)}{1+{\left(\frac{K_{a}}{{\left[Ca^{2+}\right]}_{i}}\right)}^{4}+{\left(\frac{{\left[Ca^{2+}\right]}_{i}}{K_{b}}\right)}^{3}}\right)},$$ (23)

and where *w* ^∞^ is the RyR sensitivity function

$$w^{\infty }=\left(\frac{1+{\left(\frac{K_{a}}{{\left[Ca^{2+}\right]}_{i}}\right)}^{4}+{\left(\frac{{\left[Ca^{2+}\right]}_{i}}{K_{b}}\right)}^{3}}{1+\frac{1}{K_{c}}+{\left(\frac{K_{a}}{{\left[Ca^{2+}\right]}_{i}}\right)}^{4}+{\left(\frac{{\left[Ca^{2+}\right]}_{i}}{K_{b}}\right)}^{3}}\right),$$ (24)

and K_a_, K_b_, and K_c_ are dissociation constants. [23]

#### V Sarco/endoplasmic reticulum Ca^2+^ ATPase (SERCA) and plasma membrane Ca^2+^ ATPase (PMCA)

J_Pump_ combines the pumping functions of sarco/endoplasmic reticulum Ca^2+^ ATPase (SERCA) and plasma membrane Ca^2+^ ATPase (PMCA)

$$J_{Pump}=\frac{V_{Pump}{\left[Ca^{2+}\right]}_{i}{}^{2}}{K_{Pump}{}^{2}+{\left[Ca^{2+}\right]}_{i}{}^{2}},$$ (25)

where V_Pump_ indicates the maximum flux rate of the pumps and K_Pump_ states the [Ca^2+^]_i_ for half-maximal pumping rate.

#### VI Gap junctions (GJs)

Gap junctions (GJs) and the Ca^2+^ flux via GJs ($J_{GJ,Ca^{2+}}$) are modeled as

$$J_{GJ,Ca^{2+}}=\frac{D_{Ca^{2+}}}{A_{n-1,n}}\left({\left[Ca^{2+}\right]}_{{}_{i}n-1}-{\left[Ca^{2+}\right]}_{{}_{i}n}\right)-\frac{D_{Ca^{2+}}}{A_{n,n+1}}\left({\left[Ca^{2+}\right]}_{{}_{i}n}-{\left[Ca^{2+}\right]}_{{}_{i}n+1}\right),$$ (26)

where n is the number of the NB layer. The NB layer n receives Ca^2+^ from the previous NB layer *n* − 1 and delivers Ca^2+^ to the next NB layer *n* + 1 according to the concentration gradient. Similarly, IP_3_ flux through GJs ($J_{GJ,IP_{3}}$) is modelled as

$$J_{GJ,IP_{3}}=\frac{D_{IP_{3}}}{A_{n-1,n}}\left({\left[IP_{3}\right]}_{n-1}-{\left[IP_{3}\right]}_{n}\right)-\frac{D_{IP_{3}}}{A_{n,n+1}}\left({\left[IP_{3}\right]}_{n}-{\left[IP_{3}\right]}_{n+1}\right),$$ (27)

where n is the number of the NB layer. $D_{Ca^{2+}}$is the diffusion coefficient for Ca^2+^ and $D_{IP_{3}}$is the diffusion coefficient for IP_3_. These diffusion coefficients do not take into account the open probability, regulation, or density of the GJs as they describe the actual movement of Ca^2+^ and IP_3_ from one NB layer to the next NB layer. As an exception to other NB layers, the fluxes from MS cell to NB_1_ layer are modelled by parameters $In_{Ca^{2+}}$ and $In_{IP_{3}}$ for Ca^2+^ and IP_3_, respectively. Similarly, the fluxes from NB_10_ layer to distant cell layers are modelled with parameters $Out_{Ca^{2+}}$ and $Out_{IP_{3}}$.

Parameter A describes the area of the cell membranes connecting the neighbouring NB layers in the monolayer. The value for A is received by multiplying the area of one hexagon side, that is the length of the hexagon side (l = 7μm) times the height of the cell (h = 12μm), by the number of hexagon edges between the two NB layers as

$$A_{n\to n+1}=\left(\left(3+2(n-1)\right)6\right)lh,$$ (28)

where n is the number of the NB layer (n = 1, 2, 3….10). Each NB layer has six cells with three connecting sides and (n-1) 6 cells with two connecting sides (see Fig 1). In other words, the area (A) increases with distance from the central MS cell.

## Results

### Polarization of the ARPE-19 monolayer

**Fig 4:**
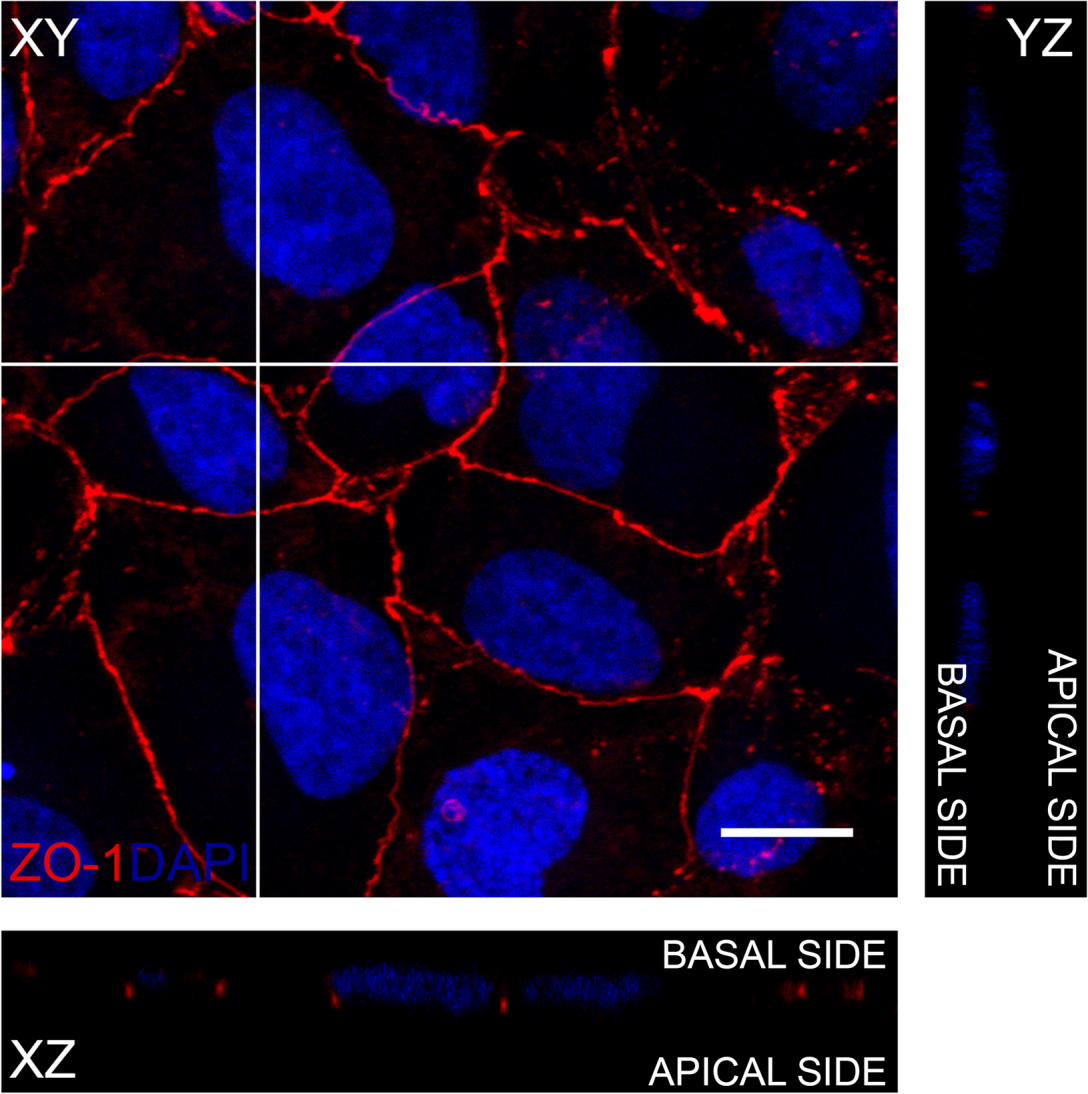
Polarization of the ARPE-19 monolayer. Z-projections (XZ and YZ) from apical side to basal side and maximum intensity projection of the XY plane in the ARPE-19 monolayer represent the localization of Zonula Occludens (ZO-1, red) in the confocal micrograph after immunofluorescence labeling with the nuclear label 4’,6-diamidino-2-phenylindole (DAPI, blue). Scale bar is 10μm.

Polarization of the ARPE-19 monolayer was demonstrated by immunolabeling the tight junctions in the monolayer. Confocal microscopy image (Fig 4) shows that within 2 days the ARPE-19 cells have formed a monolayer where ZO-1 is localised continuously in the junctions of the cells, forming a homogeneous network. This can be taken as an indication of the polarization of the epithelial cell culture [35].

### Ca^2+^ signal propagation mechanisms

**Fig 5:**
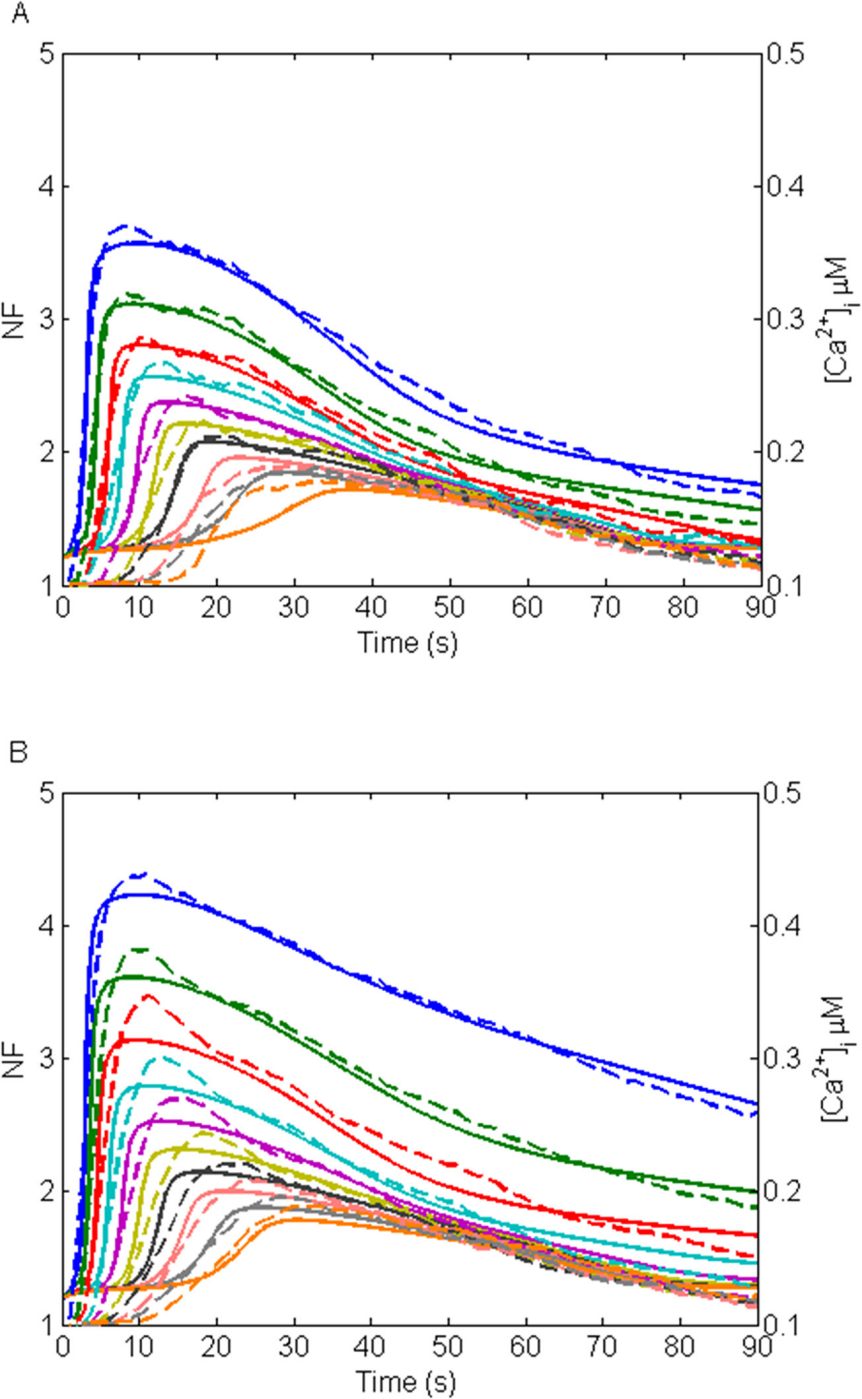
Fittings of the model to the experimental data. (A) GA-treated, and (B) control data sets with dashed lines representing the data in dimensionless NF units and solid lines representing the model simulations with arbitrary units representing [Ca^**2+**^]_i_ in μM concentrations. The uppermost curve pair (blue) represents NB1, the second uppermost NB2 (green), followed by NB3 (red), NB4 (light blue), NB5 (purple), NB6 (yellow), NB7 (black), NB8 (light red), NB9 (grey), and NB10 (orange).

The fittings of the model to the experimental data in the NB1-NB10 layers are illustrated in Fig 5A for the GA-treated data set and in Fig 5B for the control data set. The model simulations managed to catch very well the features of the experimental data in both data sets. In GA-treated data set (Fig 5A), the simulations closely followed the data in peak amplitude, time to peak, Ca^2+^ wave width at half maximum and end Ca^2+^ concentration in NB1-NB9 layers. In NB10 layer, however, time to peak was longer in the simulation results than in the data. In the control data set (Fig 5B), the Ca^2+^ wave features differed slightly between the model and the data, but overall the curve shape of the model followed the data reasonably well. R^2^ values describing the goodness of fit are presented in Table 4. In GA-treated data set and control data set R^2^ values were higher than 0.8 in NB1-NB9 and lower than 0.8 in NB10. Hence, 90% of the fits in GA-treated data set and control data set resulted in R^2^ > 0.8.

**Table 4:** R^2^ values indicating the goodness of fit between the model and the data.

Data set	NB1	NB2	NB3	NB4	NB5	NB6	NB7	NB8	NB9	NB10
GA-treated	0.9839	0.9815	0.9811	0.9651	0.9716	0.9627	0.9677	0.9326	0.8837	0.4561
Control	0.9665	0.9613	0.9454	0.9653	0.9686	0.9558	0.9508	0.9419	0.9108	0.6259
GA-suramin-treated	0.8633	0.8543	0.9344	0.9323	0.9414	0.9156	0.9253	0.7812	0.6110	0.2297

R^2^ values are listed separately for each data set and NB layer.

The model includes the model components of SSCCs, P_2_Y_2_ receptors, IP_3_R_3_s, RyRs, Ca^2+^ pumps and GJs, and the parameters were either obtained from previous studies or defined in this study for ARPE-19. The basic fit was done in GA-treated data set for NB5, but the SSCC model component was fitted in NB1 (Table 2). Three location-specific parameters were defined in this study: stretch (θ), extracellular ligand concentration (L) and phosphorylation rate of IP_3_R_3_ (α4) (Table 3). The stretch (θ) and extracellular ligand concentration (L) decayed exponentially from NB1 towards the distant NB cell layers. The IP_3_R_3_ phosphorylation rate regulated by the kinase activity (α_4_) increased following a shallow exponential, almost linear function, from NB1 to NB10. The corresponding values of α_4_ with the distance were lower in the control data set (Eq 5) than in the GA-treated data set (Eq 4) indicating a possible role of IP_3_ receptor phosphorylation rate as a regulator of Ca^2+^ signaling. The GJ model component was parameterized in control data set for NB1. GJs mediated the Ca^2+^ signal by allowing the diffusion of Ca^2+^ and IP_3_ between adjacent cell layers so that the fluxes of these species decreased with distance from the MS cell due to the increasing area of the cell membranes connecting the NB layers (Table 3).

The resulting model of mechanical stimulus induced Ca^2+^ dynamics is: 1) Cells near the stimulus site conduct Ca^2+^ through plasma membrane SSCCs, and gap junctions conduct the Ca^2+^ and IP_3_ between cells further away from stimulated cell. 2) The MS cell secretes one or several types of ligand to the extracellular space where the ligand diffusion mediates the Ca^2+^ signal so that the ligand concentration decreases with distance. 3) The phosphorylation of the IP_3_ receptor defines the cell’s sensitivity to the extracellular ligand attenuating the Ca^2+^ signal in the distance.

### Results of the sensitivity analysis

**Fig 6:**
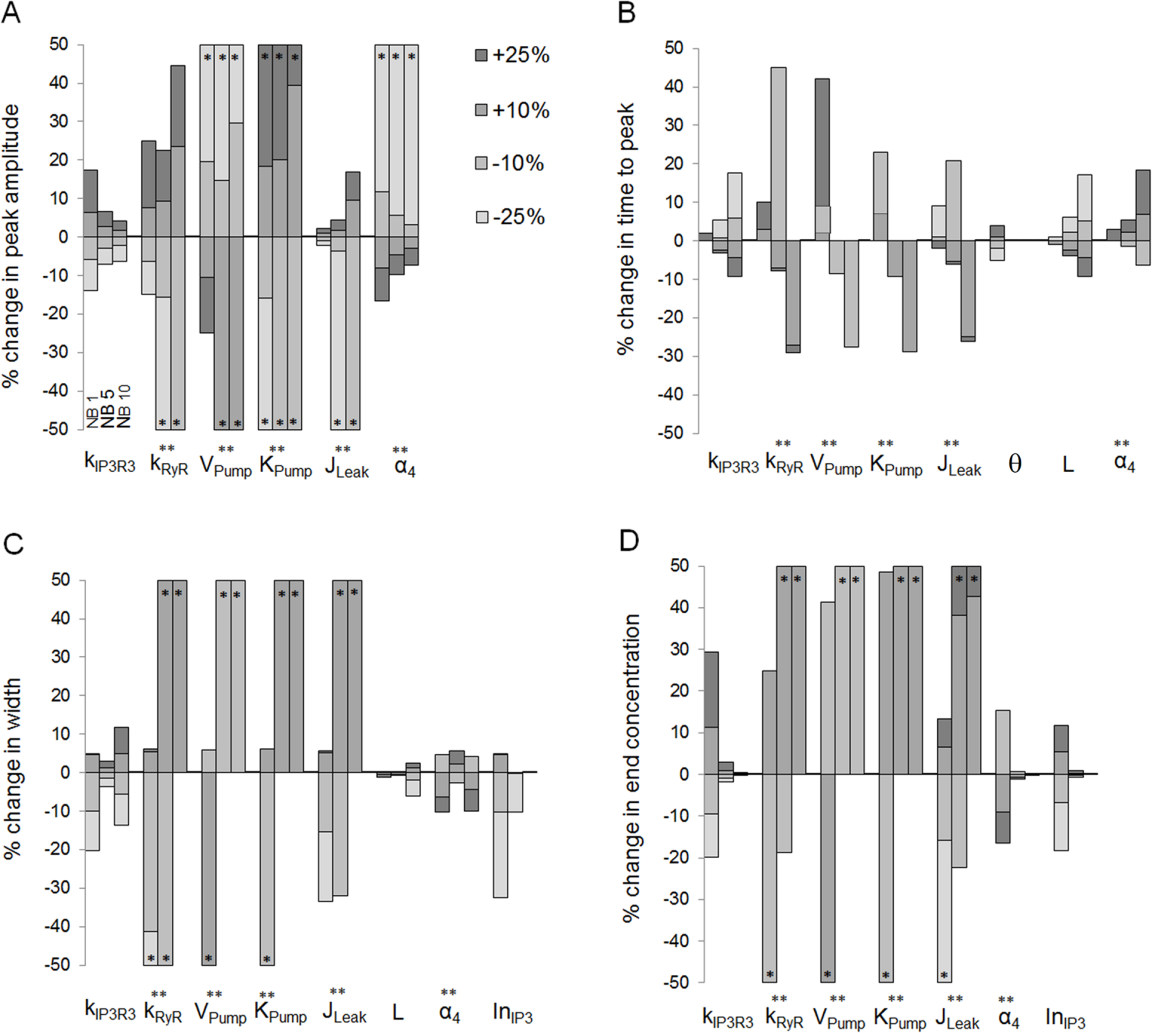
Sensitivity analysis of the model parameters. Percentage changes in Ca^**2+**^ wave (A) peak amplitude, (B) time to peak, (C) Ca^**2+**^ wave width at half maximum, and (D) end Ca^**2+**^ concentration at 90 seconds’ time point due to changes in model parameters $k_{IP_{3}R_{3}}$, k_RyR_, V_Pump_, K_Pump_, J_Leak_, θ, L, α_4_, $In_{IP_{3}}$, $In_{Ca^{2+}}$, $D_{IP_{3}}$, and $D_{Ca^{2+}}$as marked in the x-axis. The analysis was carried out in NB layers NB1, NB5 and NB10 as denoted in (A) for parameter $k_{IP_{3}R_{3}}$. The amount of modification (-25%, -10%, +10% or +25%) is shown in the grayscale of the histogram. The histogram bar is marked with asterisk (*) if the change was greater than 50%, and with double asterisk (**) if parameter modification did not result typical Ca^**2+**^ waveform. Regarding each Ca^**2+**^ wave feature, the parameter is illustrated only if the change was more than 5%.

The sensitivity of the four Ca^2+^ wave features described in Materials and Methods was studied for a set of parameters that were fitted in this study for NB1, NB5 and NB10 layers (Fig 6). From the location-dependent parameters, θ, L and α_4_, Ca^2+^ wave features were most sensitive to modifications in α_4_ and the least sensitive to modifications in θ. Overall, decreasing the stretch parameter (θ) resulted in faster Ca^2+^ waves in NB1 (Fig 6B). Increasing the extracellular ligand concentration (L) in turn decreased the time to peak (Fig 6B) and increased the Ca^2+^ wave width at half maximum (Fig 6C). The effects of the changes in IP_3_ receptor phosphorylation rate (α_4_) on Ca^2+^ wave features were complex: with decreasing α_4_ Ca^2+^ wave peak amplitude increased (Fig 6A), time to peak decreased (Fig 6B), the Ca^2+^ wave width at half maximum increased or decreased depending on the NB layer (Fig 6C), and the end concentration increased (Fig 6D). The Ca^2+^ wave features were insensitive to gap junction related parameters $In_{Ca^{2+}}$, $D_{IP_{3}}$ and $D_{Ca^{2+}}$ so that the tested modifications in their values resulted in less than 5% change in the features from the original conditions. Thus, they are not illustrated in Fig 6. However, increasing IP_3_ input to NB1 via GJs ($In_{IP_{3}}$) increased the Ca^2+^ wave width at half maximum (Fig 6C) and end concentration (Fig 6D), and the sensitivity was significantly higher in NB1 layer than in the more distant NB layers. In general, the sensitivity of the model to the changes in tested parameters depended on the NB layer and thus on the distance to the MS cell. Few parameters, however, were independent of the location (parameters $k_{IP_{3}R_{3}}$, k_RyR_, V_Pump_, K_Pump_, J_Leak_), but changes in their values affected significantly the investigated Ca^2+^ wave features.

### Possible suramin effect on attenuation of Ca^2+^ waves

**Fig 7:**
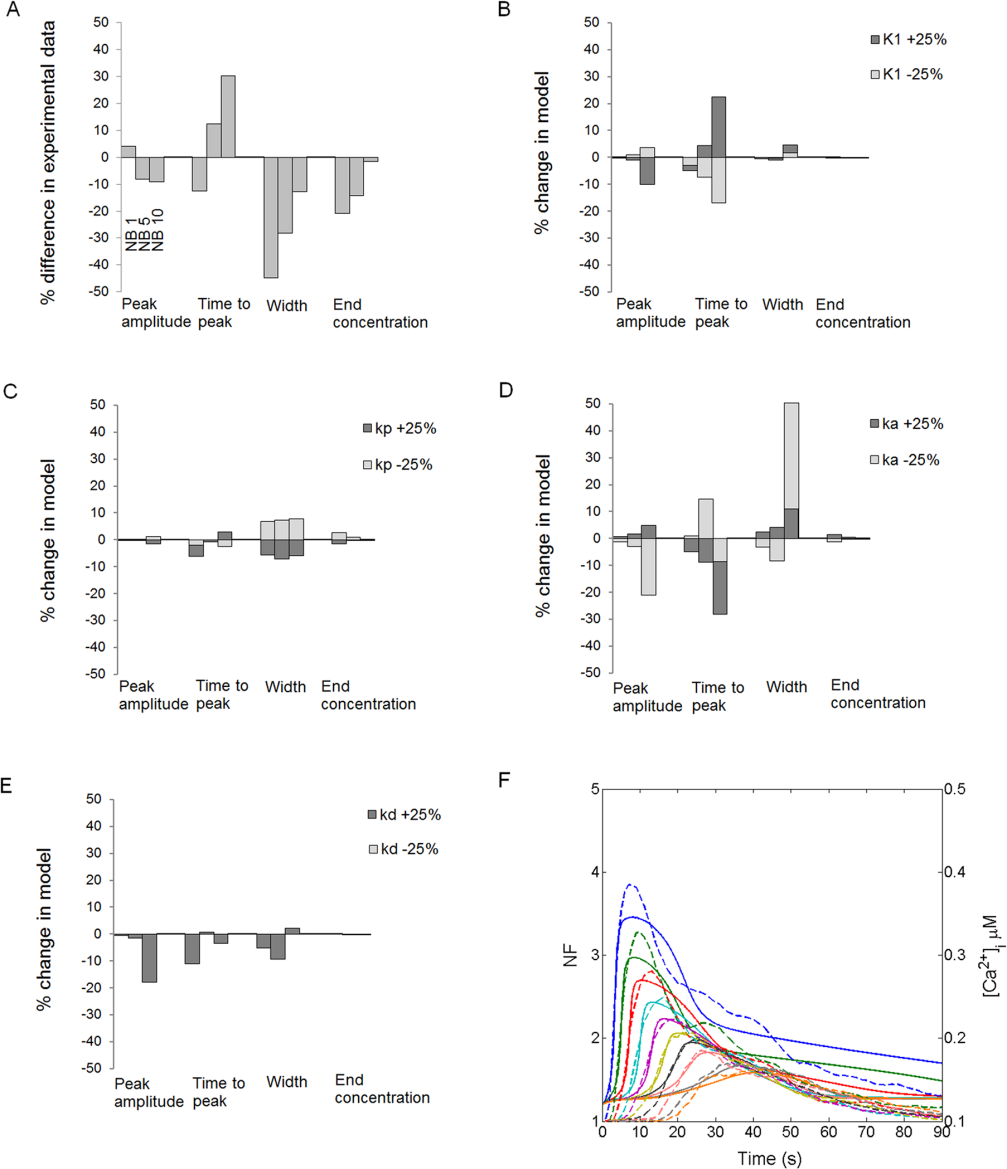
Suramin effects on Ca^2+^ wave. (A) Comparison of the peak amplitude, time to peak, Ca^**2+**^ wave width at half maximum, and end Ca^**2+**^ concentration at 90 seconds’ time point in cell layers NB1, NB5 and NB10 between the experimental GA-treated and GA-suramin-treated data sets. The deviations of each Ca^**2+**^ wave feature in GA-suramin-treated data set from GA-treated data set are expressed as percentages. (B) Unphosphorylated P_2_Y_2_ receptor dissociation constant (K_1_), (C) P_2_Y_2_ receptor phosphorylation rate (k_p_), (D) G-protein activation rate (k_a_), and (E) G-protein deactivation rate (k_d_) were changed in the model for GA-treated data set either -25% or +25%, as denoted in the grayscale of the histogram, and the percentage change in each Ca^**2+**^ wave feature is illustrated. (F) Fitting of the model to the GA-suramin-treated data set. Dashed lines represent the data in dimensionless NF units, whereas solid lines represent the model simulations with arbitrary units representing [Ca^**2+**^]_i_ in μM concentrations. The uppermost curve pair (blue) represents NB1, the second uppermost NB2 (green), followed by NB3 (red), NB4 (light blue), NB5 (purple), NB6 (yellow), NB7 (black), NB8 (light red), NB9 (grey), and NB10 (orange).

Similarly to the general sensitivity analysis, the four Ca^2+^ wave features were compared between the experimental GA-treated and GA-suramin-treated data sets as well in NB1, NB5 and NB10 layers (Fig 7A). In the GA-suramin-treated data set, differences in peak amplitude were less than 10% compared to the GA-treated data set. However, time to peak decreased for NB1 and increased for NB5 and NB10 layers in the GA-suramin-treated data set. Furthermore, in this data set the Ca^2+^ wave width at half maximum and the end Ca^2+^ concentration were lower than in the GA-treated data set.

Since suramin is a known P_2_ receptor blocker, we studied the sensitivity of the model to P_2_Y_2_ receptor parameters for the GA-treated data set (including model components I-V) by changing their values by ±25%. Similarly, G-protein cascade parameters were studied as well to consider the possible effect of suramin to disrupt the coupling between the receptor in the cell membrane and the G-protein. Our aim was to investigate the degree to which the observed differences in the Ca^2+^ wave features between GA-treated and GA-suramin-treated data sets could be accounted for by the changes in these parameters. The sensitivity analysis revealed that the modifications in P_2_Y_2_ unphosphorylated receptor dissociation constant (K_1_) and P_2_Y_2_ receptor phosphorylation rate (k_p_) indeed induced changes that were similar to the experimental observations (see Fig 7A): increase in K_1_ modified the time to peak (Fig 7B) and increase in k_p_ narrowed the Ca^2+^ wave width at half maximum (Fig 7C). This would indicate disrupted ligand binding to the receptor or a higher phosphorylation rate of P_2_Y_2_ receptors as well as a faster desensitization of the receptors after ligand binding. P_2_Y_2_ receptor parameters K_2_, k_r_, k_e_, ξ, on the contrary, had a negligible influence on Ca^2+^ wave behaviour: the modifications of these parameters by ±25% resulted only in less than 3% change on Ca^2+^ wave features, as was the case also for G-protein cascade parameter δ. G-protein cascade parameters G-protein activation rate (k_a_) (Fig 7D) and G-protein deactivation rate (k_d_) (Fig 7E) had diverse effects on the Ca^2+^ wave features: for example decreasing k_a_ and increasing k_d_ narrowed the Ca^2+^ wave width at half maximum in NB1 and NB5, but widened it in NB10. Thus, their behaviour did not follow the observations from the experimental data and therefore these factors were not considered to be responsible for the effects of suramin on the Ca^2+^ wave.

Fig 7F illustrates the fit of the model to the GA-suramin-treated data set after refitting the model parameters K_1_ and k_p_. The values of these parameters ranged as follows: K_1_ values decreased from 8.83μM in NB1 to 5.15μM in NB10 and k_p_ values decreased from 0.19s^-1^ in NB1 to 0.05s^-1^ in NB10. Overall, the values of K_1_ and k_p_ were higher in the GA-suramin-treated data set than in the GA-treated data set. The simulated curves seem to fit well to the experimental data, except in NB1 layer at the end of the Ca^2+^ wave. In GA-suramin-treated data set, R^2^ values were higher than 0.8 in NB1-NB7 and lower than 0.8 in NB8-NB10 (Table 4). 70% of the fits in GA-suramin-treated data set resulted in R^2^ > 0.8 indicating that the model explains only partially the combined effect of GA and suramin on Ca^2+^ waves especially in the distant NB layers.

## Discussion

The ARPE-19 cell line is an important biological model of human RPE despite its certain limitations [20]. This paper presents the first computational RPE model of Ca^2+^ signaling using the experimental data measured from the ARPE-19 monolayer after mechanical stimulation. We aimed to create a model that combines the most important Ca^2+^ signaling mechanisms in ARPE-19 cells so that the model can be used later in the development of more complicated RPE and epithelial models. Furthermore, the model was used to simulate and explain the Ca^2+^ signaling of epithelia, especially RPE, taking into account the following factors: 1) cells are on the monolayer; 2) they are connected to each other by GJs permeating Ca^2+^ and IP_3_, and 3) the cells are most probably experiencing different stretching and chemical conditions depending on their distance from the mechanical stimulation site. To the best of our knowledge, this is the first time as Ca^2+^ signaling model has been implemented for the ARPE-19 monolayer. The model uses a set of location specific parameters including stretch, extracellular ligand concentration, and IP_3_R_3_ phosphorylation rate as well as the Ca^2+^ and IP_3_ fluxes through GJs.

### The identity of the extracellular ligand

The airway epithelium secretes the signal carriers ATP or UTP to the extracellular space in response to mechanical stimulation[16,36]. The connection of these ligands to Ca^2+^ signaling as extracellular signal mediators has been mathematically modeled[16]. It is likely that a similar function can be linked to ARPE-19 or RPE, where the ligand interacts with the cell membrane P_2_Y_2_ receptors. In our model, the ligand carried the signal in the extracellular space from the MS cell towards the distant NB cell layers after mechanical stimulation. According to our model, the extracellular ligand concentration decreased exponentially from NB1 towards NB10. We suggest, based on our modeling results, that the MS cell secretes ligand to the extracellular space. Epithelial cells such as ARPE-19 have been shown to secrete ATP under different stimuli[37,38]. On the other hand, the ligand degradation by ectonucleotidase activity[39] decrease the ligand concentration. The model predicts that the magnitude of the extracellular ligand concentration partly defines the nature of the cell response: higher and faster Ca^2+^ waves were observed with higher ligand concentrations. The ligand concentration was derived from diffusion equation, and the obtained exponential decay function fitted well to the experimental data. Experimental studies show that the Ca^2+^ wave peak amplitude value increases with increased ligand concentration in cultured human RPE[30] and in human airway epithelium[6]. Also, in the mathematical model of Warren et al. 2010[16], it was observed that the time to peak for human airway epithelium decreased as the ligand concentration increased. These observations are in good agreement with our model.

### The role of IP_3_ receptor phosphorylation rate

The phosphorylation of the IP_3_ receptor represents an important regulatory mechanism for Ca^2+^ release[40–42]. It has been shown that the production of cyclic AMP (cAMP) through the activation of the adenylyl cyclase pathway leads to the activation of protein kinase A that phosphorylates IP_3_ receptors[43].

Our simulation results show that the maximal phosphorylation rate of IP_3_R_3_ (α_4_) followed a shallow exponential, almost linear, increase from NB1 to NB10 in all three data sets. The parameter α_4_ has previously been modeled as agonist specific only [22]. It is of note, however, that in addition to ATP or UTP and their interaction with P_2_Y_2_ receptors, also other types of ligand-receptor interactions may occur. One plausible explanation could be that MS cell secretes different types of ligands, because its cell membrane was broken in mechanical stimulation. This would further lead to complex biological interactions at the cellular level, which is seen as a chance of this parameter with cell location.

The need to model α_4_ separately for the GA-treated data set and the control data set may be related to the functioning of the GJs, especially to their ability to alter ligand secretion in different cell types. Previous studies show that GJs participate in the regulation of the release of signaling molecules to the extracellular medium [44]. In astrocytes, as an example, GJs have been proposed to regulate the release of glutamate[45], an excitatory neurotransmitter and an important regulator of astrocyte Ca^2+^ oscillations[46].

Overall, α_4_ parameter may reflect a number of ligands and cell mechanisms not modelled in this nor other epithelial Ca^2+^ models. The low α_4_ values near the MS cell enable higher and faster Ca^2+^ waves at corresponding ligand concentrations compared to the distal cell layers, where higher levels of kinase activity attenuate and slow down the signal. This aligns well with the literature. In RPE, the addition of 8-Br-cAMP counteracted the elevation of [Ca^2+^]_i_ induced by connective tissue growth factor (CTGF)[47], and the cell migration inhibitor adrenomedullin increased intracellular cAMP and decreased [Ca^2+^]_i_[48]. The effect of the adenylyl cyclase pathway on IP_3_R kinetics has been ignored in most of the previously published Ca^2+^ models e.g.[16,21,29], possibly because the kinase activity may not have been activated in those cell types or experimental conditions.

### Gap junctions in Ca^2+^ wave propagation

GJs connect the adjacent cells together and allow the diffusion of signaling molecules between them. The diffusion through GJs has previously been modeled, for example, in airway epithelium[16]. In our model, GJs carried the Ca^2+^ signaling molecules between the NB layers based on the Ca^2+^ and IP_3_ concentration gradients, and permeated Ca^2+^ and IP_3_ selectively. As expected, NB layers near the MS cell were more sensitive to IP_3_ input than the distant NB layers, and this was seen especially in the end Ca^2+^ concentration at 90 seconds’ time point.

### Possible Ca^2+^ wave attenuation mechanisms of suramin

In the GA-suramin-treated data set, the experimental data was reproduced in our model by increasing the unposphorylated receptor dissociation constant, which likely reflects disrupted ligand binding, and by increasing the phosphorylation rate of the P_2_Y_2_ receptors to enhance their desensitization. This may indicate that suramin targets on P_2_Y_2_ receptors as an unspecific P_2_ receptor antagonist attenuating the Ca^2+^ wave. This intriguing model hypothesis driven from the model results needs to be confirmed experimentally. It is worth noting, however, that suramin has also been considered to disrupt the coupling between the receptor in the cell membrane and the G-protein by blocking the association of the G-protein α and βγ subunits[33]. In our model, modifications in G-protein cascade parameters influenced the peak amplitude, time to peak and Ca^2+^ wave width at half maximum. Despite the observed diversity in their effects between the NB layers, it is possible that suramin targets the G-protein cascade as well, by acting as an attenuator of the Ca^2+^ wave.

### Limitations of the model

Our work presents a computational model of epithelial Ca^2+^ signaling based on experimental work on the ARPE-19 cell line. This cell line is used extensively as a model of RPE, although it differs from it to some extent. The limitations of ARPE-19 compared to native human RPE arise, for example, from cell organization and metabolism[20]. Importantly for our study, ARPE-19 cell line in our experimental setup lacked pigmentation which resulted in a lack of the large Ca^2+^ stores, melanosomes, and needs to be taken into account when expanding our model to describe native RPE. In addition, we confirmed the polarity of the ARPE-19 monolayer with confocal microscopy. Trans-epithelial resistance (TER) that is a general measure of epithelial integrity was not measured due to technical challenges to perform the measurements on glass cover slips with our present equipment [49]. Nevertheless, the computational model created in this study describes the most important components of epithelial and ARPE-19 Ca^2+^ activity. Thus it provides a good basis to address the native RPE in the future, even though it, being based on an *in vitro* model of RPE, needs to be considered only as a model. To improve the model further, experimental data and model implementations on certain additional Ca^2+^ related mechanisms, such as P_2_X receptors[50], voltage-sensitive Ca^2+^ channels[51] and Na^+^/Ca^2+^ exchangers[52] would be well warranted. Finally, it is worth noting that the experimental work of Abu Khamidakh et al. 2013[5] did not produce absolute Ca^2+^ concentrations, and therefore our model also features only relative Ca^2+^ activity.

## Conclusions

A full mathematical understanding of RPE and epithelial Ca^2+^ signaling would allow one to simulate cellular Ca^2+^ responses under several physiological, pathological, and experimental conditions. Our present model represents significant progress towards this goal since it is able to reproduce the experimental data from an RPE type epithelium, ARPE-19 cell line, in different conditions, simulate several epithelial Ca^2+^ signaling mechanisms, and predict drug responses in the epithelia. Our future work will include further development of the model especially focusing on the role of the voltage sensitive Ca^2+^ channels in the RPE.
